# Supplementary material for: Vaccination against H5 HP avian influenza virus leads to persistent immune response in wild king penguins
Source: Nat Commun. 2026 Feb 9;17:1395. doi: 10.1038/s41467-026-69094-9 (PMC12886961; doi:10.1038/s41467-026-69094-9)
Supplement: Supplementary file 2 — Description of Additional Supplementary Files [file 41467_2026_69094_MOESM2_ESM.pdf]

Description of Additional Supplementary Files

Supplementary Data 1. Details of the vaccination experiment data regarding serological analyses at each sampling time for the two treatment groups (control/vaccinated).

Supplementary Data 2. Data on chick morphometrics.

Supplementary Data 3. Data of the seroneutralisation assay.
