# Supplementary material for: Vaccination against H5 HP avian influenza virus leads to persistent immune response in wild king penguins
Source: Nat Commun. 2026 Feb 9;17:1395. doi: 10.1038/s41467-026-69094-9 (PMC12886961; doi:10.1038/s41467-026-69094-9)
Supplement: Supplementary file 6 — Reporting Summary [file 41467_2026_69094_MOESM6_ESM.pdf]

nature portfolio

Corresponding author(s): Thierry Boulinier

Last updated by author(s): 2025/11/22

Reporting Summary

Nature Portfolio wishes to improve the reproducibility of the work that we publish. This form provides structure for consistency and transparency in reporting. For further information on Nature Portfolio policies, see our Editorial Policies and the Editorial Policy Checklist.

Statistics

For all statistical analyses, confirm that the following items are present in the figure legend, table legend, main text, or Methods section.

n/a	Confirmed	
☐	☒	The exact sample size (*n*) for each experimental group/condition, given as a discrete number and unit of measurement
☐	☒	A statement on whether measurements were taken from distinct samples or whether the same sample was measured repeatedly
☐	☒	The statistical test(s) used AND whether they are one- or two-sided<br>*Only common tests should be described solely by name; describe more complex techniques in the Methods section.*
☒	☐	A description of all covariates tested
☐	☒	A description of any assumptions or corrections, such as tests of normality and adjustment for multiple comparisons
☐	☒	A full description of the statistical parameters including central tendency (e.g. means) or other basic estimates (e.g. regression coefficient) AND variation (e.g. standard deviation) or associated estimates of uncertainty (e.g. confidence intervals)
☐	☒	For null hypothesis testing, the test statistic (e.g. *F*, *t*, *r*) with confidence intervals, effect sizes, degrees of freedom and *P* value noted<br>*Give P values as exact values whenever suitable.*
☒	☐	For Bayesian analysis, information on the choice of priors and Markov chain Monte Carlo settings
☐	☒	For hierarchical and complex designs, identification of the appropriate level for tests and full reporting of outcomes
☒	☐	Estimates of effect sizes (e.g. Cohen's *d*, Pearson's *r*), indicating how they were calculated

Our web collection on statistics for biologists contains articles on many of the points above.

Software and code

Policy information about availability of computer code

Data collection	*Provide a description of all commercial, open source and custom code used to collect the data in this study, specifying the version used OR state that no software was used.*
Data analysis	*Provide a description of all commercial, open source and custom code used to analyse the data in this study, specifying the version used OR state that no software was used.*

For manuscripts utilizing custom algorithms or software that are central to the research but not yet described in published literature, software must be made available to editors and reviewers. We strongly encourage code deposition in a community repository (e.g. GitHub). See the Nature Portfolio guidelines for submitting code & software for further information.

Data

Policy information about availability of data

All manuscripts must include a data availability statement. This statement should provide the following information, where applicable:

- Accession codes, unique identifiers, or web links for publicly available datasets
- A description of any restrictions on data availability
- For clinical datasets or third party data, please ensure that the statement adheres to our policy

We provide in Supplementary Data 1 details of the vaccination experiment data regarding serological analyses at each sampling time for the two treatment groups

(control/vaccinated), in Supplementary Data 2 the data on chick morphometrics, and in Supplementary Data 3 data of the seroneutralisation assay. Moreover, a single source data (Excel) file is provided with all data used for the figures and analyses.

Research involving human participants, their data, or biological material

Policy information about studies with human participants or human data. See also policy information about sex, gender (identity/presentation), and sexual orientation and race, ethnicity and racism.

Reporting on sex and gender	*Use the terms sex (biological attribute) and gender (shaped by social and cultural circumstances) carefully in order to avoid confusing both terms. Indicate if findings apply to only one sex or gender; describe whether sex and gender were considered in study design; whether sex and/or gender was determined based on self-reporting or assigned and methods used. Provide in the source data disaggregated sex and gender data, where this information has been collected, and if consent has been obtained for sharing of individual-level data; provide overall numbers in this Reporting Summary. Please state if this information has not been collected. Report sex- and gender-based analyses where performed, justify reasons for lack of sex- and gender-based analysis.*
Reporting on race, ethnicity, or other socially relevant groupings	*Please specify the socially constructed or socially relevant categorization variable(s) used in your manuscript and explain why they were used. Please note that such variables should not be used as proxies for other socially constructed/relevant variables (for example, race or ethnicity should not be used as a proxy for socioeconomic status). Provide clear definitions of the relevant terms used, how they were provided (by the participants/respondents, the researchers, or third parties), and the method(s) used to classify people into the different categories (e.g. self-report, census or administrative data, social media data, etc.) Please provide details about how you controlled for confounding variables in your analyses.*
Population characteristics	*Describe the covariate-relevant population characteristics of the human research participants (e.g. age, genotypic information, past and current diagnosis and treatment categories). If you filled out the behavioural & social sciences study design questions and have nothing to add here, write "See above."*
Recruitment	*Describe how participants were recruited. Outline any potential self-selection bias or other biases that may be present and how these are likely to impact results.*
Ethics oversight	*Identify the organization(s) that approved the study protocol.*

Note that full information on the approval of the study protocol must also be provided in the manuscript.

Field-specific reporting

Please select the one below that is the best fit for your research. If you are not sure, read the appropriate sections before making your selection.

☐ Life sciences ☐ Behavioural & social sciences ☒ Ecological, evolutionary & environmental sciences

For a reference copy of the document with all sections, see nature.com/documents/nr-reporting-summary-flat.pdf

Life sciences study design

All studies must disclose on these points even when the disclosure is negative.

Sample size	*Describe how sample size was determined, detailing any statistical methods used to predetermine sample size OR if no sample-size calculation was performed, describe how sample sizes were chosen and provide a rationale for why these sample sizes are sufficient.*
Data exclusions	*Describe any data exclusions. If no data were excluded from the analyses, state so OR if data were excluded, describe the exclusions and the rationale behind them, indicating whether exclusion criteria were pre-established.*
Replication	*Describe the measures taken to verify the reproducibility of the experimental findings. If all attempts at replication were successful, confirm this OR if there are any findings that were not replicated or cannot be reproduced, note this and describe why.*
Randomization	*Describe how samples/organisms/participants were allocated into experimental groups. If allocation was not random, describe how covariates were controlled OR if this is not relevant to your study, explain why.*
Blinding	*Describe whether the investigators were blinded to group allocation during data collection and/or analysis. If blinding was not possible, describe why OR explain why blinding was not relevant to your study.*

Behavioural & social sciences study design

All studies must disclose on these points even when the disclosure is negative.

Study description	*Briefly describe the study type including whether data are quantitative, qualitative, or mixed-methods (e.g. qualitative cross-sectional, quantitative experimental, mixed-methods case study).*
Research sample	*State the research sample (e.g. Harvard university undergraduates, villagers in rural India) and provide relevant demographic*

Research sample	*information (e.g. age, sex) and indicate whether the sample is representative. Provide a rationale for the study sample chosen. For studies involving existing datasets, please describe the dataset and source.*
Sampling strategy	*Describe the sampling procedure (e.g. random, snowball, stratified, convenience). Describe the statistical methods that were used to predetermine sample size OR if no sample-size calculation was performed, describe how sample sizes were chosen and provide a rationale for why these sample sizes are sufficient. For qualitative data, please indicate whether data saturation was considered, and what criteria were used to decide that no further sampling was needed.*
Data collection	*Provide details about the data collection procedure, including the instruments or devices used to record the data (e.g. pen and paper, computer, eye tracker, video or audio equipment) whether anyone was present besides the participant(s) and the researcher, and whether the researcher was blind to experimental condition and/or the study hypothesis during data collection.*
Timing	*Indicate the start and stop dates of data collection. If there is a gap between collection periods, state the dates for each sample cohort.*
Data exclusions	*If no data were excluded from the analyses, state so OR if data were excluded, provide the exact number of exclusions and the rationale behind them, indicating whether exclusion criteria were pre-established.*
Non-participation	*State how many participants dropped out/declined participation and the reason(s) given OR provide response rate OR state that no participants dropped out/declined participation.*
Randomization	*If participants were not allocated into experimental groups, state so OR describe how participants were allocated to groups, and if allocation was not random, describe how covariates were controlled.*

Ecological, evolutionary & environmental sciences study design

All studies must disclose on these points even when the disclosure is negative.

Study description	In this study, we report the results of a vaccination trial using a saRNA vaccine against H5 HPAI clade 2.3.4.4b protein conducted on king penguin chicks in a sub-Antarctic colony of the Southern Indian Ocean (Possession Island, Crozet Archipelago, 46°25'34.23"S; 51° 51'39.97"E). A group of 30 chicks received a primo-injection at about 45 days post-hatching (once emancipated from their parents), and a booster 37 days later, while a group of 20 chicks was used as controls. Along 250 days of fine-scale monitoring, the vaccinated individuals showed a high and persistent anti-H5 immune response, granting a strong seroneutralisation capacity against a H5N1 2.3.4.4b clade virus, up to fledging. We report no adverse effects due to the vaccination treatment on chick health and survival. The screening for antibodies against unspecific avian influenza viruses suggested that no natural infection occurred across the entire trial, although the end of the experiment overlapped with the local emergence of HPAI H5N1 in October 202414, highlighting the timeliness of such an experiment. Overall, our results suggest that the saRNA H5 vaccine could be a valuable and powerful tool for wildlife conservation efforts in the current context of panzootic HPAI.
Research sample	30 vaccinated individuals and 20 control individuals.
Sampling strategy	Repeated temporal sampling along the chick rearing period. Samples sizes for vaccinated and control individuals were pre-determined according to basic power calculations and the expected immune responses and potential mortalities along the chick rearing period.
Data collection	Blood sampling at times of vaccine injection and at a series of 5 recaptures.
Timing and spatial scale	252 days during the rearing pearing period of king penguin chicls, with start of the experiment in March 2024 and end of the experiment in November 2024.
Data exclusions	None
Reproducibility	Single run of the experiment.
Randomization	Random attribution of the individuals to the vaccination versus control group.
Blinding	Blinding for recaptures.

Did the study involve field work? ☒ Yes ☐ No

Field work, collection and transport

Field conditions	Field work conducted on île de la Possession, Crozet archipelago, Southern Indian Ocean?
Location	Crozet Archipelago, 46°25'34.23"S; 51°51'39.97"E
Access & import/export	Import permit : DDPP 2024276 ; Permit to conduct experiment from TAAF authority : TAAF 2024-07 ; and from Ministry of Research : APAFIS #45548-2023102712468223 v3

Disturbance	Limited disturbance, with work under permit from the National Nature Reserve Authority (Prefecture TAAF).

Reporting for specific materials, systems and methods

We require information from authors about some types of materials, experimental systems and methods used in many studies. Here, indicate whether each material, system or method listed is relevant to your study. If you are not sure if a list item applies to your research, read the appropriate section before selecting a response.

Materials & experimental systems

n/a	Involved in the study
☐	☒ Antibodies
☒	☐ Eukaryotic cell lines
☒	☐ Palaeontology and archaeology
☐	☒ Animals and other organisms
☒	☐ Clinical data
☒	☐ Dual use research of concern
☒	☐ Plants

Methods

n/a	Involved in the study
☒	☐ ChIP-seq
☒	☐ Flow cytometry
☒	☐ MRI-based neuroimaging

Antibodies

Antibodies used	Anti-H5 and anti-NP Avian Influenza commercial ELISA were used, from Innovative Diagnostics (Grabels, France).
Validation	The competitive ELISA used are validated for all species according to the manufacturer. The use of an indirect ELISA developed for chicken is indirectly validated by prior work (as cited in the manuscript, Garnier et al. 2017 Functional Ecology), and by the current work which shows that the results of the two ELISAs are very similar (Figure 3 presents the results of the competitive anti-H5 ELISA and Supplementary figure 1, the results of the indirect anti-H5 ELISA).

Animals and other research organisms

Policy information about studies involving animals; ARRIVE guidelines recommended for reporting animal research, and Sex and Gender in Research

Laboratory animals	No
Wild animals	Yes, king penguin chicks _Apptenodytes patagonicus_
Reporting on sex	No sexing (both sex included)
Field-collected samples	Yes. All field collected blood samples, on heparinized serynge, with dcentrifugation conducted in the field to store plasma.
Ethics oversight	Evaluation by the regional ethical commitee of Occitanie Est and by Conseil de l'Environnement Polaire.

Note that full information on the approval of the study protocol must also be provided in the manuscript.

Plants

Seed stocks	*Report on the source of all seed stocks or other plant material used. If applicable, state the seed stock centre and catalogue number. If plant specimens were collected from the field, describe the collection location, date and sampling procedures.*
Novel plant genotypes	*Describe the methods by which all novel plant genotypes were produced. This includes those generated by transgenic approaches, gene editing, chemical/radiation-based mutagenesis and hybridization. For transgenic lines, describe the transformation method, the number of independent lines analyzed and the generation upon which experiments were performed. For gene-edited lines, describe the editor used, the endogenous sequence targeted for editing, the targeting guide RNA sequence (if applicable) and how the editor was applied.*
Authentication	*Describe any authentication procedures for each seed stock used or novel genotype generated. Describe any experiments used to assess the effect of a mutation and, where applicable, how potential secondary effects (e.g. second site T-DNA insertions, mosiacism, off-target gene editing) were examined.*
